# Annotations

**Published:** 1908-02-01

**Authors:** 


### Annotations.

### Medical Women and the Royal Colleges.

Some months ago, it will be remembered, a petition, promoted by the London School of Medicine for Women, was presented to the Royal College of Physicians and to the Royal College of Surgeons praying that the examinations of these colleges should be thrown open to women. Later it was announced that the Council of the Royal College of Surgeons had passed a resolution in favour of admitting women to the examinations which qualify for the membership, but reserving the Fellowship for men only. The College of Physicians has taken even a more liberal view, and has expressed its readiness to admit women to all its examinations. At the same time, however, the College has recorded its determination not to take any action under this resolution inconsistent with the agreement existing between the two Colleges. There are two circumstances which render this expression of policy of great moment. The first is that the two Colleges are bound by a common understanding not to confer their minimum qualifications—the Licence in the one case, and the Membership in the other—apart from one another. In the second place, the Council of the College of Surgeons has determined, before putting the decision into action, to take a poll of the Fellows and Members. Presumably, if the result is adverse, the Council will withdraw the resolution in favour of the admission of women, and in these circumstances the vote of the Royal College of Physicians, in consequence of the agreement between the two corporations, will be of no avail, so far, that is, as the M.R.C.S. and the L.R.C.P. qualifications are concerned. There remains the M.R.C.P., which, of course, rests entirely with the College of Physicians. Thus the decision on the most practical portion of the women's petition rests with the Fellows and Members of the Royal College of Surgeons, and it remains to be seen whether they will or will not endorse the decision of their Council.

### The late Sir Thos. McCall Anderson, M.D.

It is with much regret that we announce the death of Sir Thos. McCall Anderson, Regius Professor of Medicine in the University of Glasgow. Born in 1836, the late Professor was, during the whole of his life, closely associated with the University and Medical School of his native city, and his death removes one of the recognised leaders of the profession in the West of Scotland. Besides his professorship, Sir Thomas was Honorary Physician to the King, and he held the position of Senior Physician to the Glasgow Western Infirmary and to the Hospital for Diseases of the Skin; he was also the representative of the University on the General Medical Council. His career as a teacher extends over some forty years. After graduation and a prolonged term of study on the Continent, he returned to Glasgow, and was soon elected to the Chair of Medicine in Anderson's College and as a physician on the staff of the Royal Infirmary. When the University buildings were moved to Gilmorehill, and the Western Infirmary was founded, Dr. McCall Anderson was appointed a physician and clinical teacher in the Infirmary, and in 1874 he became Professor of Clinical Medicine in the University. This office he held until 1900, when he succeeded the late Sir Wm. Gairdner as Regius Professor of Medicine. To the profession generally Sir Thomas is perhaps best known by his text-book and other publications on diseases of the skin, and more recently by his repeated advocacy of the use of tuberculin in cases of phthisis pulmonalis and other tubercular affections. As a teacher he commanded large classes of pupils, and this more particularly in the hospital wards and in his clinique for diseases of the skin; his orderly, systematic, and punctual methods constituted a useful and effective discipline in the training of the medical student. His extensive professional experience and his unfailing courtesy made him a welcome consultant both to the public and to his *confrères*, and his death will cause general and sincere regret.

### The Ophthalmo-Tuberculin Reaction.

Professor Calmette has published further experiences of the application of a solution of tuberculin to the conjunctiva as a means of diagnosis in cases of suspected tubercular disease. In every one of 321 patients suffering from one or other clinical manifestation of the effects of tubercle a positive reaction was obtained. It is also stated that a positive reaction was obtained in some patients who were under treatment for non-tuberculous complaints, and that on closer examination almost all of these were found to have suspicious signs of lesions either in the lymphatic glands or in the lungs. An important question in connection with this test is whether a response may be due to the presence of a healed tubercular lesion in one or other part of the body. Were this so the value of the test as a method of diagnosis in the presence of active symptoms having more or less uncertain significance would be seriously limited, as a positive reaction would not necessarily mean that such symptoms were dependent on a tubercular process—the reaction, in other words, might be the expression, not of the existing active disease, but of some previously exhausted tubercular activity. At present it does not seem possible to say that this uncertainty can be resolved either one way or the other, but it is at least satisfactory to have Professor Calmette's assurance that in none of his cases did the test produce any harmful effect on the lesions from which the patient was known to be suffering. He also states that in no instance was there any rise of temperature or other evidence of general disturbance. Hence it may be fairly concluded that there is no risk attached to the method beyond the slight inconvenience associated with the conjunctivitis which is the sign of a positive result. It may be noted, too, that the degree of reaction is quite independent of the size of the lesion—a limited affection of the lymph-glands may lead to a more definite response than a case of phthisis with much cavitation. Altogether this test, though not perhaps absolute, is a very valuable addition to our diagnostic resources in cases of suspected tubercle.

